# A Physiologically Based Pharmacokinetic (PBPK) Modeling Framework for Mixtures of Dioxin-like Compounds

**DOI:** 10.3390/toxics10110700

**Published:** 2022-11-17

**Authors:** Rongrui Liu, Tim R. Zacharewski, Rory B. Conolly, Qiang Zhang

**Affiliations:** 1 Lower Merion High School, Ardmore, PA 19003, USA; 2 Department of Biochemistry and Molecular Biology, Institute for Integrative Toxicology, Michigan State University, East Lansing, MI 48824, USA; 3 Ramboll US Consulting, Inc., Monroe, LA 71201, USA; 4 Gangarosa Department of Environmental Health, Rollins School of Public Health, Emory University, Atlanta, GA 30322, USA

**Keywords:** dioxin-like compounds, mixture, TCDD, PBPK, AHR, TEQ

## Abstract

Humans are exposed to persistent organic pollutants, such as dioxin-like compounds (DLCs), as mixtures. Understanding and predicting the toxicokinetics and thus internal burden of major constituents of a DLC mixture is important for assessing their contributions to health risks. PBPK models, including dioxin models, traditionally focus on one or a small number of compounds; developing new or extending existing models for mixtures often requires tedious, error-prone coding work. This lack of efficiency to scale up for multi-compound exposures is a major technical barrier toward large-scale mixture PBPK simulations. Congeners in the DLC family, including 2,3,7,8-tetrachlorodibenzo-p-dioxin (TCDD), share similar albeit quantitatively different toxicokinetic and toxicodynamic properties. Taking advantage of these similarities, here we reported the development of a human PBPK modeling framework for DLC mixtures that can flexibly accommodate an arbitrary number of congeners. Adapted from existing TCDD models, our mixture model contains the blood and three diffusion-limited compartments—liver, fat, and rest of the body. Depending on the number of congeners in a mixture, varying-length vectors of ordinary differential equations (ODEs) are automatically generated to track the tissue concentrations of the congeners. Shared ODEs are used to account for common variables, including the aryl hydrocarbon receptor (AHR) and CYP1A2, to which the congeners compete for binding. Binary and multi-congener mixture simulations showed that the AHR-mediated cross-induction of CYP1A2 accelerates the sequestration and metabolism of DLC congeners, resulting in consistently lower tissue burdens than in single exposure, except for the liver. Using dietary intake data to simulate lifetime exposures to DLC mixtures, the model demonstrated that the relative contributions of individual congeners to blood or tissue toxic equivalency (TEQ) values are markedly different than those to intake TEQ. In summary, we developed a mixture PBPK modeling framework for DLCs that may be utilized upon further improvement as a quantitative tool to estimate tissue dosimetry and health risks of DLC mixtures.

## 1. Introduction

Dioxin-like compounds (DLCs) are a group of structurally similar persistent organic pollutants (POPs), including compounds in the families of polychlorinated dibenzo-p-dioxins (PCDDs), polychlorinated dibenzofurans (PCDFs), and polychlorinated biphenyls (PCBs). In general, DLCs contain seven 2,3,7,8–substituted PCDDs, ten 2,3,7,8–substituted PCDFs, and twelve non-ortho- or mono-ortho-substituted PCBs, which share a common mechanism of toxicities [1,2]. PCDD/Fs are produced in a number of industrial and natural processes, including steel smelting, automobile exhaust, incineration, such as municipal waste and forest burning, paper production, and as byproduct during the synthesis of organic compounds, such as herbicides [3]. PCDD/Fs can be dispersed into the soil, water, and air, and then spread to the general population. PCBs are produced for usage in coolants, electrical insulators, capacitors, and carbonless copy papers [4]. These DLCs have been at the top of man-made emissions and identified in at least 126 National Priorities List (NPL) hazardous waste sites in the US [5].

DLCs are highly toxic and can cause a variety of adverse health outcomes, including cancer, developmental defects, immunotoxicity, metabolic and endocrine disorders [1]. 2,3,7,8-Tetrachlorodibenzo-p-dioxin (TCDD) is the most toxic congener, and the remaining DLCs are generally less toxic. By activating the aryl hydrocarbon receptor (AHR), DLCs share a common mode of action. Humans are exposed to environmental pollutants existing in mixtures. While it is important to understand and estimate the health risk of individual DLCs, such as the reference compound TCDD or the most prevalent congeners, it is the mixtures in the environment that ultimately drive the AHR-mediated health outcomes. Environmental exposures to DLC mixtures have been linked to many health endpoints. For instance, analyzing the data from the 1999–2004 cycles of the National Health and Nutrition Examination Survey (NHANES), Lin et al. showed a significant positive association between all-cause mortality risk and the toxic equivalent (TEQ) values of serum DLC mixtures including 7 PCDDs, 10 PCDFs, and 9 PCBs [6]. Similarly, using the 1999–2002 cycles of NHANES, significant negative associations were found between serum total T4 and TEQ of 7 PCDDs, 5 PCDFs, and 4 PCBs, especially in older women [7]. In the longitudinal Russian Children’s Study, the serum TEQ of seven serum PCDDs was found to be associated with lower sperm concentration, sperm count, and total motile sperm count in young men [8]. Higher cancer-related mortality was suggested for residents near a Waelz plant where the air PCDD/Fs levels were high [9]. Therefore, developing the capability of making accurate predictions of the health risk of exposure to DLC mixtures has significant public health implications.

The compositions of DLC mixtures in the environment are highly variable. For instance, those at the Superfund sites vary greatly in the types of constituent congeners and concentrations in the soil sediments within and between polluted sites [10,11]. The compositions of DLC mixtures that humans are exposed to, mainly through dietary intake by consuming fish and fatty meats, are also highly variable [12,13,14,15,16]. While concentrations in the sources or doses in the exposures provide some preliminary information on the toxicity potentials of DLC mixtures, they are still far removed from the apical health endpoint. More proximal to the biological effects, human blood or tissue concentrations, available through biomonitoring [9,17,18], autopsy [19] or in silico predictions, are the preferred metrics used for estimating the health risk of mixtures.

Physiologically based pharmacokinetic (PBPK) models can quantitatively predict the relationship between the exposures and tissue concentrations by mathematically describing the physiological and biochemical processes involved in the absorption, distribution, metabolism, and elimination (ADME) of chemicals. Over the past three decades, there has been a rich literature on dioxin PBPK modeling for species including rodents and humans on various physiological stages including developmental, lactational, lifetime, and obesity [15,20,21,22,23,24,25,26,27,28,29]. The vast majority of these studies were focused on TCDD, and the four-compartment model developed by Emond et al. has been adopted by US EPA for dioxin risk assessment [30]. Nevertheless, these models are for single-congener exposures only, even in studies where a number of congeners were covered [15,29]. In general, adapting an existing PBPK model developed for one specific compound to a structurally similar compound in the same family is straightforward, since the same model structure can be used and for the most part it involves estimating chemical-specific parameters for the new compound. If at the exposure levels of concern there are no congener–congener interactions, e.g., the congeners do not compete for binding to kinetically important proteins or enzymes or they do not cross-induce common metabolic enzymes, then modeling the mixtures can be carried out simply by simulating each congener independently.

A dioxin mixture PBPK model does not fall into this case. The pharmacokinetics of DLCs is nonlinear and dose-dependent, primarily due to processes in the liver that are specific to dioxins [31,32]. First, a dioxin compound can bind to the CYP1A2 protein in the liver such that its free concentration is greatly reduced due to the sequestration by the protein. Second, through binding to and thus activating AHR, a dioxin compound can induce the gene transcription of CYP1A2, which further enhances the sequestration of the compound and also the clearance of dioxin where CYP1A2 acts as the main metabolic enzyme [33]. As a result, for a DLC mixture exposure, different congeners will compete for binding to CYP1A2 and also collectively induce it to sequester and metabolize one another. Therefore, significant nonlinear congener–congener interactions could exist in the toxicokinetic process, and simulating each congener individually and subsequently combining the results linearly may not make accurate predictions of the mixture effect. In this situation, all congeners in a mixture have to be simulated simultaneously in an integrated mixture PBPK model.

Traditionally, developing a mixture model would require manually extending the existing PBPK model constructed for a single compound to include new ordinary differentiation equations (ODEs) that would track the concentrations of new compounds added in the mixture and adjusting the existing ODEs for common variables, such as AHR and CYP1A2 in the case of DLC mixture, accordingly. This type of manual programming is tedious and error-prone and is not flexible as more compounds are considered. The lack of flexibility to efficiently scale up for multi-compound exposures constitutes a major technical barrier toward large-scale mixture simulations. The main goal of the present study is to develop a coding scheme or framework for DLC mixture PBPK models that can flexibly accommodate an arbitrary number of congeners without requiring manual modification of the model. The mixture model was constructed by adapting a TCDD model previously developed by Emond et al. [23,28].

To estimate the toxicity potencies of DLCs relative to TCDD, toxic equivalency factors (TEFs) have been established for the congeners based on expert evaluations of a multitude of in vivo and in vitro experimental studies [34]. With the TEF of TCDD at 1, the TEFs currently adopted for other congeners range from 0.00003 to 1 with half order-of-magnitude increments [2,35]. To quantify the toxicity potentials of a DLC mixture in exposure, TEQ values are calculated using the TEF-weighted sum of DLC doses. Although the TEQ approach was developed with the intention to estimate the toxicity potential of oral exposure to DLC mixtures, the approach is also used often on the source matrix at polluted sites to compare the degrees of contamination at different locations [11].

It is important to note that a potential pitfall in the TEQ approach is that most of the “intake” TEF values were derived primarily based on in vivo rodent studies and thus their direct applications to humans are not without uncertainties given very different rodent vs. human toxicokinetics of DLCs including half-lives [36]. Moreover, with the assumption of concentration addition, these TEFs were established based on single exposure experiments, not mixtures [2]. Therefore, using exposure or intake doses to calculate TEQ only treats these compounds independently, without taking into consideration the potential toxicokinetic inter-dependence or interactions of DLCs. It has been recommended that calculating body burden TEQ, by developing and applying “systemic” TEFs based on in vitro assays to biomonitoring data or model-predicted plasma or tissue concentrations of DLCs, will help in reducing the inter-species uncertainty in toxicokinetics and improving the accuracy of the TEQ approach for human risk assessment [2,37]. Therefore, developing a human DLC mixture PBPK model that can predict the internal doses of congeners will help toward this goal.

## 2. Methods

### 2.1. Model Structure

**Figure 1:**
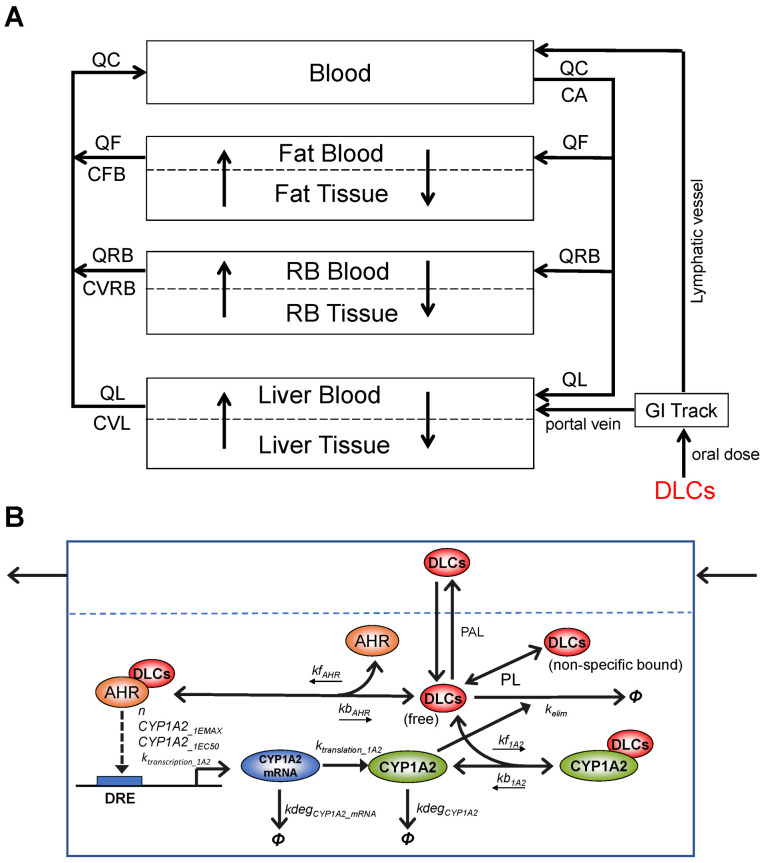
**Schematic illustration of the structure and molecular key events of the DLC mixture PBPK model.** (**A**) The overall structure of the DLC mixture PBPK model containing four blood circulation-connected compartments: *Blood*, *Fat*, *RB*, and *Liver*. The latter three are further divided into blood and tissue subcompartments to account for diffusion-limited distribution processes. (**B**) Details of the molecular key events in *Liver Tissue*: Competitive, reversible binding of DLCs for AHR (parameterized with *kf_AHR_* and *kb_AHR_*) and CYP1A2 (*kf_1A2_* and *kb_1A2_*), transcriptional induction of CYP1A2 mRNA by DLCs-AHR complexes governed by a Hill function (*n*, *CYP1A2__1EMAX_*, *CYP1A2__1EC50_*, *k_transcription_1A2_*), degradation of CYP1A2 mRNA (*kdeg_CYP1A2_mRNA_*), protein translation of CYP1A2 (*k_translation_1A2_*), degradation of CYP1A2 (*kdeg_CYP1A2_*), CYP1A2-mediated elimination of DLCs (*k_elim_*), and fast equilibrium between free and non-specific bound fractions of DLCs (as defined by the partition coefficients PL). *Φ* denotes degradation or elimination. Open arrow head: flux, solid arrow head: regulation.

We constructed the human DLC mixture model by adapting a TCDD PBPK model developed by Emond et al. The Emond model was first constructed for rats [24,25], by simplifying a more complex model originally developed by Wang [21]. Then, the model was modified through multiple iterations for humans [23,26,28] and mice [27]. The Emond model has been adopted by US EPA for dioxin risk assessment [30]. The Emond model basically consists of four tissue compartments: *Liver*, *Fat*, rest of the body (*RB*), and *Blood*, each connected by the systemic circulation (Figure 1A). The dioxin distributions in *Fat*, *Liver*, and *RB* compartments are described as diffusion-limited processes. When only oral exposure is considered, the up-taken dioxin compound goes into the *Liver* through the portal vein and *Blood* through the lymphatic vessels. In the *Liver*, TCDD binds reversibly to both AHR and CYP1A2, and TCDD-liganded AHR can induce production of CYP1A2, which in turn sequesters and also metabolizes TCDD. While human CYP1A1 and CYP1B1 showed high activities toward low-chlorinated PCDDs, their activities toward TCDD and other 2,3,7,8–substituted PCDD/Fs are largely negligible (Van den Berg et al. 1994, Inui et al. 2014). Therefore, specific metabolism mediated by CYP1A1 and CYP1B1 was not considered in the Emond model and nor in our mixture model. Nevertheless, our model did inherit from the Emond model a slow first-order process of clearance of dioxin from the blood, which can be considered as accounting for other non-CYP1A2-mediated metabolism activities.

While maintaining the overall structure of the Emond TCDD model, we adapted it for the DLC mixture model with some modifications and assumptions of the molecular toxicodynamic events in the *Liver Tissue*. **(1)** In the original model, the free TCDD concentration in the *Liver Tissue* was not treated as a state variable that is described by an ODE. It is solved analytically as the root of a quadratic function that is derived based on the quasi-steady state assumption for the binding between TCDD and AHR and CYP1A2 following the law of mass action and conservation [24,25,28]. However, with the mixture model where multiple DLC congeners compete for AHR and CYP1A2, it is no longer tractable, if not entirely possible, to solve the liver free concentration of each congener analytically. Instead, in our mixture model, we describe explicitly the association and dissociation between each congener and AHR/CYP1A2, as illustrated in Figure 1B, and the free and nonspecific bound concentration of each congener is tracked by an ODE and solved numerically. **(2)** DLC congeners can be different in their AHR binding affinities but have the same CYP1A2 induction efficacy, i.e., at saturating concentrations, each congener can independently induce CYP1A2 to the same maximal abundance. This assumption is consistent with the TEF concept, i.e., DLC congeners differ in relative potency, but have the same efficacy in toxicity [2]. **(3)** The Emond model uses two intermediate CYP1A2 species to provide some time delay for the induction of CYP1A2 protein by TCDD-liganded AHR. While the approach serves its purpose, the molecular nature of the two intermediates is not entirely clear. In our mixture model, we replaced the two intermediates with the mRNA species of CYP1A2 (Figure 1B). While this modification is trivial, it makes the model more aligned with the molecular biology of gene induction. This delay becomes rather insignificant when the model is simulated for lifetime exposure to DLCs.

### 2.2. Automated Vector Representation of Congener ODEs

Many modern programming languages, such as MATLAB, Octave, R, Python, and Julia, have the built-in capability of matrix manipulation, which can be utilized to simplify the construction of a mixture PBPK model for congeners that share common ADME mechanisms. This capability would save us from manually writing out the same ODE set for each new congener added to a mixture. This is realized by expressing the set of ODEs describing the time derivatives of the amounts of the congeners in a tissue compartment in a vector format. Here, we present an example for DLC congeners in *Fat Blood* (Equation (1)) and *Fat Tissue* (Equation (2)):

(1) $$\frac{dy}{dt}\;\left(N+4\;:\;L\;:\;N+\left(M-1\right)\cdot L+4\right)=QF*\left(CA-\frac{AFB}{VFB}\right)-PAF⨀\left(\frac{AFB}{VFB}-\frac{AF}{VF}⨀\frac{1}{PF}\right),$$

(2) $$\frac{dy}{dt}\;\left(N+5\;:\;L\;:\;N+\left(M-1\right)\cdot L+5\right)=PAF⨀\left(\frac{AFB}{VFB}-\frac{AF}{VF}⨀\frac{1}{PF}\right),$$

where ⨀ denotes element-wise vector multiplication, *M*, *N*, and *L* are the number of congeners, number of repeating state variables, and starting index of the ODE vector, respectively. *PAF* and *PF* are congener-specific parameters in vector format with length of *M* for the permeability area cross product in *Fat* and fat:blood partition coefficient, respectively. *CA*, *AFB,* and *AF* are state variables in vector format with length of *M* for arterial concentrations, amounts in *Fat Blood*, and amounts in *Fat Tissue,* respectively. Finally, *VFB* and *VF* are scalar physiological parameters for the volumes of *Fat Blood* and *Fat Tissue,* respectively.

The ODE of free CYP1A2 protein in the *Liver Tissue* is presented below as an example of common state variables shared by all congeners:

(3) $$\begin{array}{c} \frac{dy}{dt}\;\left(N+1+M\cdot L\right)=k_{translation\_1A2}\cdot CYP1A2\_mRNA-kdeg_{CYP1A2}\cdot \;CYP1A2-\sum (kf_{1A2}⨀\frac{\mathrm{AL}}{\mathrm{VL}}⨀\frac{1}{\mathrm{PL}}\cdot \\ CYP1A2)+\sum \left(kb_{1A2}⨀Dioxin\_CYP1A2\right), \end{array}$$

where *CYP1A2_mRNA* and *CYP1A2* are scalar state variables for the mRNA and protein species of CYP1A2, respectively. *AL* and *Dioxin_CYP1A2* are state variables in vector format with length of *M* for the amounts of free + nonspecific bound dioxin congeners and concentrations of congener-bound CYP1A2 protein complexes, respectively. *VL* is the *Liver* volume and *PL* is the congener-specific liver:blood partition coefficient in vector format with length of *M*. *k_translation_1A2_* and *kdeg_CYP1A2_* are scalar physiological parameters for the translation and degradation of *CYP1A2,* respectively. The *kf_1A2_* and *kb_1A2_* are congener-specific parameters in vector format with length of *M* for the association rate constants and dissociation rate constants of binding of congeners to *CYP1A2*. The ∑ function is used to sum up all the association rates and dissociation rates involved in the reversible binding events between the multiple congeners in a mixture and *CYP1A2*. The three exemplary ODEs show that the vector representation of repeating state variables for congeners can scale automatically with *M*, the number of congeners in the mixture without the need of manually adding, removing or modifying ODEs. The ODEs of the mixture PBPK model are listed in Table S1.

### 2.3. Model Parameterization

*Physiological/biochemical parameters:* The physiological and most biochemical parameters of the mixture model, including tissue volumes and blood flows, remain the same as in the Emond model for humans (Table S2). The only exceptions are the biochemical parameters related to the translation (*k_translation_1A2_*) and degradation (*kdeg_CYP1A2_*) of CYP1A2 protein and the transcription (*k_transcription_1A2_*) and degradation (*kdeg_CYP1A2_mRNA_*) of CYP1A2 mRNA which replaces the two CYP1A2 intermediates as described above. These values were adjusted such that the basal and maximally induced steady-state levels of *CYP1A2* remain the same as in the Emond model. The mRNA level remained as 10% of the protein. The protein half-life, as determined by ln2/*kdeg_CYP1A2_*, was doubled to compensate for the shortened delay resulting from the replacement of the two intermediate steps with the mRNA single step. Regardless, the delay in CYP1A2 production is insignificant when the model is simulated for lifetime exposure to DLCs.

*Chemical-specific parameters:* The TCDD-specific parameters, including tissue:blood partition coefficients, elimination rate constants, and binding affinities for AHR and CYP1A2, remain the same as in the Emond model. Since explicit binding processes between TCDD and AHR and between TCDD and CYP1A2 are introduced in the mixture model as described above, new parameters are added here, including the association rate constant (*kf_AHR_*) and dissociation rate constant (*kb_AHR_*) for TCDD and AHR binding, and the association rate constant (*kf_1A2_*) and dissociation rate constant (*kb_1A2_*) for TCDD and CYP1A2 binding. The corresponding dissociation constants, i.e., *Kd_AHR_* = *kb_AHR_*/*kf_AHR_* and *Kd_1A2_* = *kb_1A2_*/*kf_1A2_*, remain the same as in the Emond model. The dissociation of liganded AHR can be slow as reported in several species where the half-life can be hours to days [38,39,40]. In comparison, the dissociation of liganded human AHR is faster, in minutes [41]. Given the lifetime exposure we considered here, the absolute value of *kb_AHR_* is not important. Therefore, we set it to 6/h, corresponding to a dissociation half-life of about 10 min. A similar principle was followed to define the parameters for CYP1A2 binding.

A total of 11 DLC congeners including TCDD are used in the present study. These compounds are top ranking congeners in the sediment and soil samples at Superfund sites near the Tittabawassee River and Saginaw bay [10,11]. For non-TCDD congeners, their dissociation constants, *Kd_AHR_*, for AHR binding were estimated by scaling the one for TCDD with the relative potency (REP) values based on the receptor binding assay if available or other in vitro assays reported in the 2004 REP database for DLCs [35]. Then, the corresponding *kf_AHR_* value for each congener was obtained by *kf_AHR_* = *kb_AHR_*/*Kd_AHR_* assuming that the *kb_AHR_* values remain the same across congeners. The binding affinities of all congeners for CYP1A2 were assumed to be the same as TCDD. The partition coefficients, *PL*, *PF*, and *PRB*, and GI track absorption rate *KST* of congeners were estimated by scaling the corresponding values of TCDD in the Emond model with the values estimated for different congeners by Maruyama et al. [15,29]. Similarly, the hepatic elimination rate constants *kelim* were estimated by scaling TCDD with the half-lives estimated for different congeners in [15]. Since urinary elimination only constitutes a tiny fraction of overall dioxin clearance as in the Emond model, the urinary excretion rate constants *CLURI* remained the same as TCDD for all congeners. Chemical-specific parameter values for all the 11 congeners are provided in Table S3. In practice, these parameter values are stored in a Microsoft spreadsheet which is automatically read into the Octave program by the PBPK model.

### 2.4. Monte Carlo Simulation

Monte Carlo simulations were conducted for 1000 human individuals with the following parameters sampled from predefined distributions. For oral exposure doses, the dietary intake values estimated for a Dutch population in 1994 were utilized [14]. Each congener’s daily exposure dose was sampled from lognormal distributions with means and coefficients of variation (CV) provided by the Dutch study (Table S4). For body weight variations, each coefficient of the 4th-order polynomial function used to describe the lifetime body weight growth in the Emond model was sampled from a lognormal distribution with the mean equal to the default coefficient value and CV of 0.01. For biochemical parameter variations, *AHR_tot_*, *k_transcription_1A2_*, and *CYP1A2_1EMAX* were selected among the top sensitive parameters based on the sensitivity analysis result of the Emond model previously reported [25,30]. These three parameters were sampled from lognormal distributions with the mean equal to their default values and CV of 0.3.

### 2.5. Calculation of TEQ

Exposure or intake TEQ is calculated by multiplying the gram dose of each DLC congener by its TEF as reported in [2] and then summing the results. Plasma or tissue TEQ is calculated by multiplying the gram concentration of each DLC congener by its human systemic TEF as estimated in [37].

### 2.6. Modeling Tools and Sharing

Open-source GNU Octave Version 6.2.0 was used to construct the DLC mixture PBPK model and the *lsode()* function was used for numerical simulation. The model code is available on GitHub at https://github.com/pulsatility/2022-DLC-Mixture-PBPK (accessed on 14 November 2022). For model comparison and validation purpose, the original Emond model was run in Berkeley Madonna (Version 8.3.18, University of California, Berkeley, CA, USA).

## 3. Results

### 3.1. Model Validation for TCDD Exposure

The original Emond models for TCDD were developed in ACSL or acslX (Aegis Corporation, Huntsville, AL, USA) with subsequent iterations for rodents and humans also available in Berkeley Madonna [28,42]. The human version of the Emond model was optimized and validated using a variety of human data [23] and has been selected by USEPA for dioxin risk assessment [30]. These datasets include plasma and/or fat concentrations in US Air Force veterans exposed to TCDD during the mission of aerial spraying of Agent Orange and herbicides from Operation Ranch Hand during the Vietnam war [43], human volunteers orally administered a single dose of TCDD [44], and patients intoxicated with TCDD exhibiting clinical chloracne [45]. Since the Emond model was extensively validated against these datasets, it follows that if our modified human model can perform the same as the Emond model, then our model is validated for TCDD exposure.

**Figure 2:**
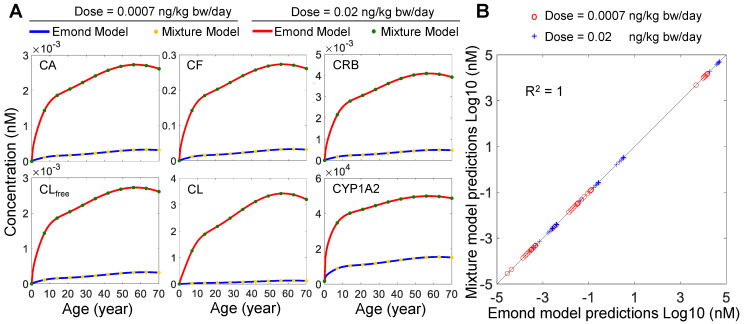
**Performance comparison of the mixture model and Emond model**. (**A**) Predicted time−course tissue concentrations of TCDD for the *Blood* (*CA*), *Fat* (*CF*), *RB* (*CRB*), and *Liver* (free: *CL_free_*, total: *CL*) compartments, and the induced liver *CYP1A2* levels for lifetime exposure to two doses by the two models as indicated. (**B**) Scatter plot of Log10-transformed tissue TCDD and *CYP1A2* concentrations at select ages (year 1, 10, 20, 30, 40, 50, 60, and 70) predicted by the two models in (**A**).

To validate the performance of our modified human mixture model for TCDD exposure only, we ran the two models (i.e., our model in Octave and the Emond model in Berkeley Madonna) for both the low-dose and high-dose lifetime exposure scenarios based on the EPA-recommended reference dose (RfD) and point-of-departure (PoD) dose [30]. The PoD dose, 0.02 ng/kg bw/day, was derived from the lowest-observed-adverse-effect levels (LOAEL) reported in two previous studies [46,47], and the RfD is 0.0007 ng/kg bw/day after applying an uncertainty factor of 30 to the PoD dose [30]. Our model predicts an essentially identical time-course accumulation of tissue concentrations of TCDD as the Emond model in the *Blood* (*CA*), *Fat* (*CF*), *RB* (*CRB*), and *Liver* (free: *CL_free_*, total: *CL*) compartments as well as the induced *Liver CYP1A2* levels for lifetime exposures to both doses in women (Figure 2A). When the tissue TCDD and *Liver CYP1A2* concentrations at select ages (year 1, 10, 20, 30, 40, 50, 60, and 70) predicted by the two models are scatter-plotted, they fall nearly perfectly on the diagonal with R^2^ = 1 (Figure 2B). The result demonstrates that the behavior of our modified model implemented in Octave is basically identical to the Emond model for both low-dose and high-dose TCDD lifetime exposures. Moreover, a similar result was obtained for men (not shown), and unless otherwise specified, the remaining simulation results are presented for women.

### 3.2. Simulations of Binary Mixtures

**Figure 3:**
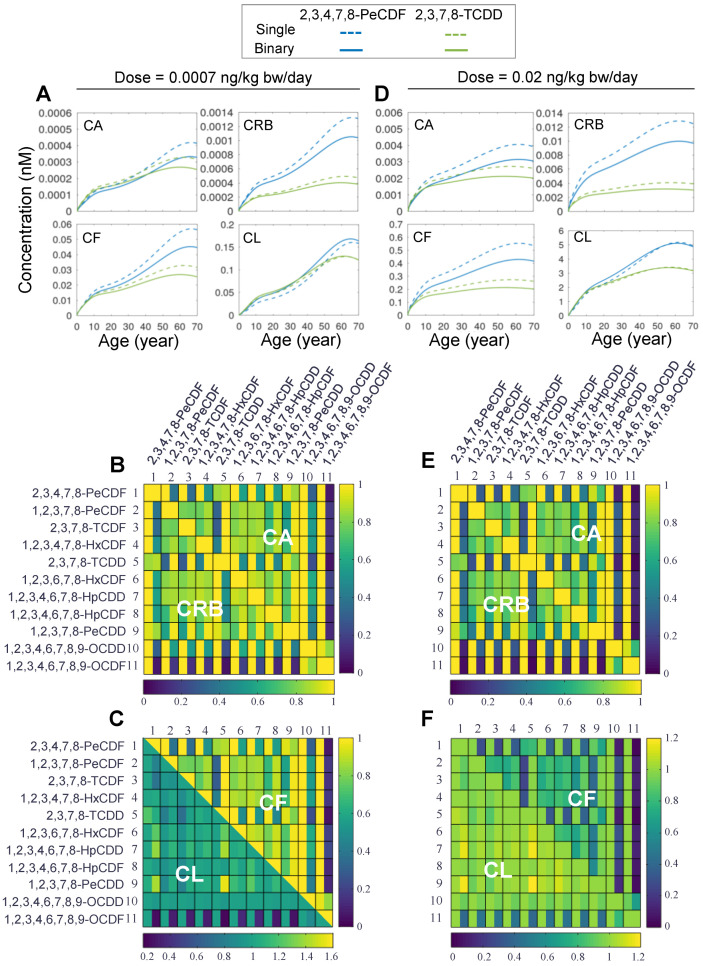
**Simulation results for binary mixture exposures to DLCs.** (**A**) Predicted time-course tissue concentrations of *CA*, *CF*, *CRB*, and *CL* for lifetime exposure to single 2,3,4,7,8-PeCDF (dashed blue line), single (dashed green line) 2,3,7,8-TCDD or binary mixture (solid blue and green lines, respectively) at low dose (0.0007 ng/kg bw/day). (**B**,**C**) Lifetime-averaged ratios (indicated by the heatmap color) of *CA*, *CF*, *CRB*, and *CL* in a binary exposure to the corresponding concentrations in single exposures for all pair-wise combinations of 11 DLCs as indicated for low-dose exposure of 0.0007 ng/kg bw/day. The diagonal represents unity. Except on the diagonal, each square contains two vertical bins each corresponding to the concentration ratio for one of the two congeners in a binary mixture. (**D**–**F**) Same as (**A**–**C**) except for high-dose exposure at 0.02 ng/kg bw/day.

Next, we set out to examine the toxicokinetics of DLC mixtures. Herein, we first considered the simple case of binary mixtures. It is expected that the cross-induction of CYP1A2 by the two congeners in a binary mixture will lead to increased sequestration of each congener and since CYP1A2 is also the metabolizing enzyme here, it will promote the hepatic clearance of both congeners. Therefore, binary mixture exposures are expected to lead to lower concentrations of the two congeners than single exposures. Time-course simulations of a representative binary mixture, 2,3,4,7,8-PeCDF and TCDD, for both the low- (Figure 3A) and high-dose (Figure 3D) exposure scenarios clearly showed that the *CA*, *CRB*, and *CF* of 2,3,4,7,8-PeCDF (solid blue) and TCDD (solid green) are all lower, to various degrees, than the corresponding concentrations (dashed blue and green, respectively) in single exposures throughout the lifetime. The total liver concentrations, *CL*, are of exception, where they are most of the time slightly higher in a binary mixture exposure than in single exposures. The increased liver burden can be explained by the fact that a mixture exposure would cause higher induction of CYP1A2, which can sequester more congeners in the liver despite the fact that their free concentrations are lower than in single exposures.

Next, we examined whether the above result that extrahepatic tissue burdens are reduced and hepatic burden is increased, obtained for the 2,3,4,7,8-PeCDF and TCDD mixture, is a general feature of binary DLC exposures. We expanded the binary mixture simulations to all other pair-wise combinations of the 11 DLC congeners for both low- (Figure 3B,C) and high-dose (Figure 3E,F) lifetime exposures. The model’s framework of vector representation of congener ODEs greatly facilitated the sweeping through all pair-wise combinations. The results indicate that the ratios of *CA*, *CRB*, and *CF* in a binary exposure to the counterpart concentrations in single exposures, averaged over lifetime, are always less than unity, as shown by the heatmaps in Figure 3B–F. For the low-dose exposure, the lowest ratio is 0.053 for the *CA* of 1,2,3,4,6,7,8,9-OCDF when it is in mixture with TCDD (Figure 3B). For the high-dose exposure, the lowest ratio is 0.03 for the *CRB* of 1,2,3,4,6,7,8,9-OCDF when it is in mixture with TCDD (Figure 3E). In contrast, the liver burden (*CL*) of a congener in a binary exposure is mostly on par with or slightly higher than that in single exposures (lower half in Figure 3C,F). For the low-dose exposure, the highest *CL* ratio is 1.55 for 1,2,3,7,8-PeCDD when it is in mixture with TCDD (Figure 3C). For the high-dose exposure, the highest *CL* ratio is 1.2, also for 1,2,3,7,8-PeCDD when it is in mixture with TCDD (Figure 3F). However, there are still cases where the liver burden is dramatically reduced when a congener is in a binary mixture. This occurs when 1,2,3,4,6,7,8,9-OCDF is in mixture with TCDD where the *CL* ratio is 0.24 for low-dose exposure (Figure 3C) and 0.4 for high-dose exposure (Figure 3F). In summary, binary DLC mixture exposures tend to reduce the extrahepatic tissue burdens of individual congeners while the liver burden may change in either directions depending on the binary combinations.

### 3.3. Simulation of High-Order Mixtures

**Figure 4:**
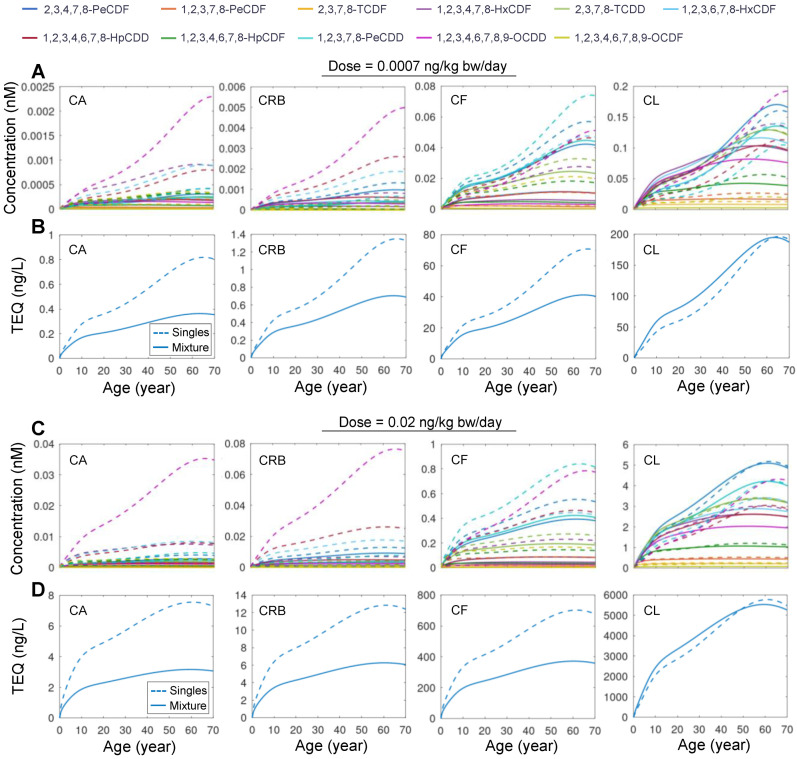
**Simulation results for mixture exposure to 11 DLCs.** (**A**) Predicted time-course of *CA*, *CF*, *CRB*, and *CL* for lifetime exposure to mixture of 11 DLCs (solid line) or single DLC (dashed line) as indicated at a dose of 0.0007 ng/kg bw/day for each congener. (**B**) Tissue TEQ values calculated based on the concentrations in (**A**) using systemic TEF. (**C**,**D**) Same as (**A**,**B**) but for high-dose exposure at 0.02 ng/kg bw/day for each congener.

Next, we examined the mixture of all 11 DLCs. The simulation showed that at equal doses of 0.0007 or 0.02 ng/kg bw/day, the *CA*, *CRB*, and *CF* of each congener in the mixture exposure are consistently lower than in single exposure (Figure 4A,C). For low-dose exposure, 1,2,3,4,6,7,8,9-OCDF has the lowest mixture:single concentration ratio, reaching 0.016 for *CA*, *CRB*, and *CF* at the age of 70 years, while 2,3,4,7,8-PeCDF has the highest ratio, reaching 0.74 at the age of 70 years. Moreover, 1,2,3,4,6,7,8,9-OCDF has the lowest mixture:single concentration ratio for *CL*, reaching 0.015 at the age of 70 years, while 1,2,3,7,8-PeCDD has the highest *CL* ratio, reaching 1.26 at the age of 70 years. For high-dose exposure, 1,2,3,4,6,7,8,9-OCDF is consistently the congener that has the lowest ratio for all concentrations, while 2,3,4,7,8-PeCDF and 1,2,3,7,8-PeCDD are also the two congeners exhibiting the highest ratios for extrahepatic and hepatic concentrations, respectively.

The health risk of DLC mixture exposure is traditionally estimated using the TEQ approach. While this method has been applied to DLC mixtures in the food consumed or even source matrix, such as soil, when tissue or plasma concentrations of DLCs are available, TEQ values derived using systemic TEF rather than the traditional “intake” TEF are believed to be a better metric for evaluating the health risk potential of DLC mixture exposures [37]. Therefore, we used the human-specific systemic TEF estimated in [37] to compare the tissue TEQ of mixture exposure vs. single exposures. For both the low- and high-dose exposure scenarios, the TEQ values calculated based on *CA*, *CRB,* and *CF* in the mixture exposure are markedly lower than the TEQ values summed across 11 single-congener exposures (Figure 4B,D). In contrast, the TEQ values for *CL* are generally higher in the mixture exposure than summed single exposures. These results demonstrated that PBPK modeling of single exposures may overestimate the extrahepatic body TEQ and health risk compared with mixture modeling.

### 3.4. Monte Carlo Simulation of Population Variability

**Figure 5:**
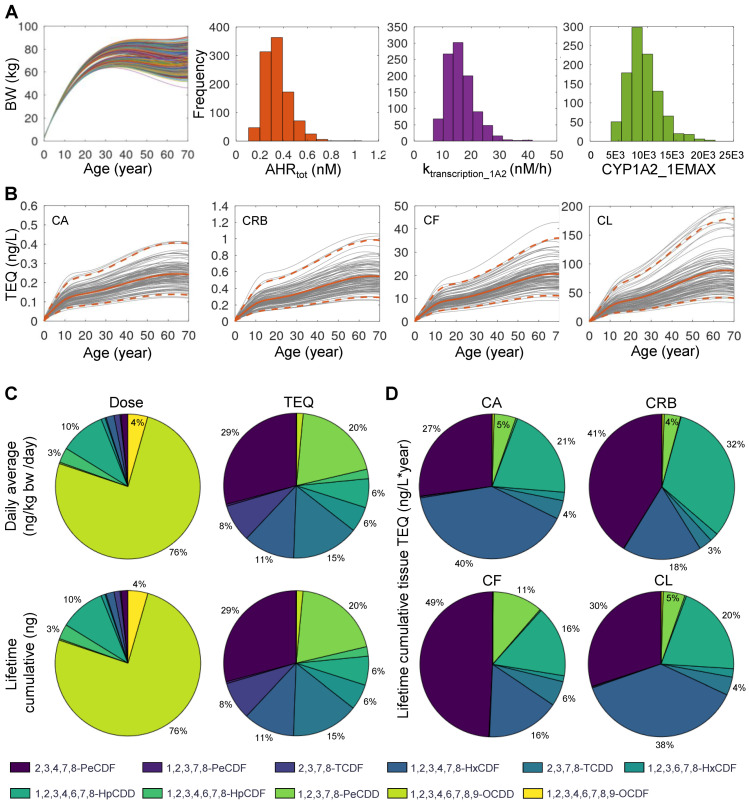
**Monte Carlo simulation of 1000 human individuals exposed to 11 DLCs.** (**A**) Inter-individual variations in lifetime body weight (BW) changes and three biochemical parameters as indicated. (**B**) The mean (orange solid line) and 2.5–97.5 percentile (orange dashed line) levels of blood or tissue TEQ as indicated, overlaid with 100 randomly selected individuals (gray lines). (**C**) Percentage contributions of individual DLC congeners to daily average mass dose (top-left) or lifetime cumulative mass dose (bottom-left) as indicated. Percentage contributions of individual DLC congeners to daily average TEQ (top-right) or lifetime cumulative TEQ (bottom-right) as indicated. (**D**) Percentage contributions of individual DLC congeners to lifetime cumulative blood or tissue TEQ as indicated. The color scheme of the congeners is indicated at the bottom.

Human individuals are exposed to DLC mixtures of different compositions and levels and they are also different in physiological and biochemical parameters related to dioxin ADME, including body weight, adipose tissue fraction, AHR activation, and CYP1A2 induction [30,48]. In this section, we aimed to illustrate the utility of the DLC mixture PBPK model in simulating a human population and comparing the contributions of individual congeners to TEQ calculated based on different dose metrics. Monte Carlo sampling of exposures to the 11 DLC congeners is described in Methods. Each human individual is assumed to be exposed to a constant dose of a congener in ng/kg bw/day through lifetime. The variations of lifetime body weight growth and the three selected biochemical parameters are presented in Figure 5A for 1000 sampled individuals where males and females were equally represented. The simulated blood and tissue TEQ values over lifetime, calculated using systemic TEF values, are shown in Figure 5B, overlaid with the population mean and 2.5 and 97.5 percentile levels. The distribution of the daily average mass dose of the 11 congeners is presented in Figure 5C (top-left panel), where OCDD contributes to 75% of the total dose followed by 1,2,3,4,6,7,8-HpCDD at 10%, OCDF at 4%, and 1,2,3,4,6,7,8-HpCDF at 3%, while the remaining congeners are negligible. When the lifetime cumulative dose of each congener in each individual is calculated, the distribution is basically identical to the daily average dose (Figure 5C, bottom-left panel). Next, we compared the contributions of individual congeners to intake TEQ. The contributions of individual congeners to daily (Figure 5C, top-right panel) or cumulative (Figure 5C, bottom-right panel) intake TEQ are markedly different than their mass dose compositions. Here, 2,3,4,7,8-PeCDF contributes the most, at 29%, followed by 1,2,3,7,8-PeCDD at 20%, TCDD at 15%, 1,2,3,4,7,8-HxCDF at 11%, 2,3,7,8-TCDF at 8%, and both 1,2,3,6,7,8-HxCDF and 1,2,3,4,6,7,8-HpCDD at 6%, while the remaining congeners are negligible.

Interestingly, for lifetime cumulative tissue-level TEQ, the relative contributions of individual congeners are dramatically different compared with their contributions to intake TEQ. The top three contributors are TCDD, 2,3,4,7,8-PeCDF, and 1,2,3,4,6,7,8-HpCDD. However, their relative importance varies in different tissues. For *CA* TEQ (Figure 5D, top-left panel), they follow the order of TCDD (40%), 2,3,4,7,8-PeCDF (27%), and 1,2,3,4,6,7,8-HpCDD (21%). For *CRB* TEQ (Figure 5D, top-right panel), the order is 2,3,4,7,8-PeCDF (41%), 1,2,3,4,6,7,8-HpCDD (32%), and TCDD (18%). For *CF* TEQ (Figure 5D, bottom-left panel), the order is 2,3,4,7,8-PeCDF (49%), and TCDD and 1,2,3,4,6,7,8-HpCDD both at 16%. Interestingly, for *CL* TEQ, the contribution of the three congeners follow the same order as *CA* TEQ: TCDD (38%), 2,3,4,7,8-PeCDF (30%), and 1,2,3,4,6,7,8-HpCDD (20%).

## 4. Discussion

PBPK modeling has been an important in silico tool in chemical safety assessment. Since humans are exposed to environmental pollutants in mixtures, it calls for PBPK models that can simulate co-exposure to multiple compounds simultaneously. When developing mixture models, it is common to start with PBPK models of single compounds and then combine them based on the known or best hypothesized compound interaction mechanisms [49,50]. Many mixture PBPK models, primarily for binary and some for higher-order mixtures, have been developed for both environmental and pharmaceutical compounds [51,52,53,54,55,56,57]. Constructing mixture PBPK models is time consuming, especially for mixtures containing diverse compounds, where pair-wise interactions can be compounds-specific. To simulate a high-order mixture, all binary interactions of the compounds in the mixture need to be characterized and appropriately captured mathematically [50,55,56,58]. Therefore, scaling up mixture PBPK models is a complex and challenging task.

Yet, for compounds belonging to the same family and sharing common toxicokinetic and toxicodynamic mechanisms, mixture PBPK modeling may be simplified, as we did with the modeling framework for DLC mixtures here. The rationale hinges on the fact that DLC congeners can induce the CYP1A2 protein through activating a common nuclear receptor AHR, and CYP1A2 then sequesters and metabolizes the congeners. The DLC mixture model was based on an early human PBPK model for single TCDD exposure [25,28]. A major modification made to the Emond model is on how the DLC binding to AHR and CYP1A2 is modeled. In early days of PBPK modeling when the computation resource and power was limiting, a common practice was that fast processes, such as binding between chemicals and proteins, were often assumed at quasi-steady state relatively to other slower ADME processes, such that analytically solvable algebraic equations rather than ODEs were used to track the fast-reacting species to speed up the numerical integration. While this analytical approach is manageable for modeling one or two congeners, it quickly becomes intractable as more congeners are added to the mixture, which all compete for binding to AHR and CYP1A2. To circumvent this problem, we resorted to modeling these reversible binding processes by describing both the association and dissociation steps explicitly. While this modification may slow down the simulation to some extent, it provides the freedom to include an arbitrary number of DLC congeners into the mixture model.

For mixture PBPK modeling, the amount of coding generally increases linearly with the number of compounds in the mixture since the tissue concentrations of each compound need to be tracked separately. In the present study, we utilize a vector to hold the ODEs describing the concentrations of all DLC congeners in a particular tissue rather than hard coding each congener separately. In addition, as detailed in the ODEs in Table S1, vectors are used conveniently to sum for the total amounts of DLC-bound AHR and DLC-bound CYP1A2, and for the total rates of association and dissociation for the binding processes, as done in the ODEs of common state variables AHR and CYP1A2. This vector-based approach not only saves the extra error-prone coding work, but also provides the flexibility to scale with the number of congeners in the mixture through varying the vector length accordingly. Although our model is implemented in Octave, it can be easily adapted to run in any other modern programming languages that can handle matrices, including R, Python, and Julia. Sasso et al. proposed a similar matrix-based approach for metals and nonmetal mixture modeling using a general model structure [59]. However, the chemical interactions in the mixtures still need to be handled on a case-by-case basis.

The biggest uncertainty in the DLC mixture model lies in the chemical-specific parameters associated with the non-TCDD congeners, which have no validated, preexisting PBPK models similar to the TCDD model. Following the underlying principle in the TEQ approach, we assumed that the DLC congeners differ in their potency of AHR activation (i.e., *Kd_AHR_*) but are equally efficacious in their transcriptional activity to induce CYP1A2. While this is likely true for most of the congeners, it may not apply to all congeners [60]. Nevertheless, this assumption of equal efficacy reduces the number of chemical-specific parameters and simplifies the coding for AHR-mediated induction of CYP1A2. The *Kd_AHR_* value is not available for every congener. While some were calculated based on the REP values of receptor binding assays, many had to be estimated based on other in vitro assays, as annotated in Table S3. Another uncertainty is with CYP1A2 binding. Without prior information, we had to assume that every congener has the same binding affinity for CYP1A2 as TCDD. This assumption will clearly affect their hepatic burden predicted by the PBPK model. Finally, the hepatic elimination rate constant of each congener was estimated based on their reported half-lives and scaled with TCDD. Despite these uncertainties, it is worth noting that constructing a rigorously parameterized DLC mixture model is not the main purpose of the present study, rather, we aim to develop a mixture modeling framework for DLCs which can be further refined with better informed congener-specific parameter values in the future.

As a starting point, some of the assumed or uncertain congener-specific parameters, such as binding affinities for AHR and CYP1A2 may be predicted using in silico tools, such as quantitative structure-activity relationship (QSAR) modeling followed by confirmation with measurements using binding assays [61,62]. Human cells-based in vitro assays can be utilized to measure hepatic enzymatic activities that can be extrapolated to in vivo conditions to parameterize liver clearance for the PBPK model [63,64]. Yet, validating the PBPK model for each non-TCDD DLC would require comprehensive datasets including (1) longitudinal human blood and tissue concentrations of DLCs, such as in fat and liver, and (2) related exposure data or estimation, albeit simultaneously obtaining both on the same individuals can be challenging. Structurally, the DLC PBPK model can be improved by including dose-independent pathways of elimination and reducing the dose-dependent elimination as suggested in [30]. As lipophilic compounds, the bound vs. unbound fractions of TCDD and other DLCs in the plasma can be significant, but they are not distinguished in the Emond and other dioxin models. Given the important role of plasma unbound fraction in chemical toxicokinetics, future iteration of the model should consider these factors explicitly. The present modeling framework is still steps away from application for DLC mixture risk assessment. In addition to rigorously optimizing and validating the model using parameter, exposure, and tissue burden data as described above, inter-individual variabilities need to be considered. These variabilities will include physiological variations in body weight growth and fat fractions, polymorphism in AHR abundance and affinity, and CYP1A2 induction and enzymatic activities [48,65,66]. Finally, a hierarchical Bayesian PBPK modeling approach can be utilized to integrate the population and individual-level parameter as well as exposure variabilities and uncertainties to better characterize the tissue TEQ burdens for mixture risk assessment [67].

Utilizing the mixture PBPK model, we found that due to the cross-induction of CYP1A2, the extrahepatic tissue burden of a congener in mixture exposure is always lower than in single exposure. In contrast, the hepatic burden can increase or decrease depending on the compositions of the mixture. If the two congeners are both strong CYP1A2 inducers, such as TCDD and 1,2,3,7,8-PeCDD, their hepatic burdens tend to increase due to more sequestration by CYP1A2. If one congener is significantly stronger than the other in inducing CYP1A2, such as TCDD and 1,2,3,4,6,7,8,9-OCDF, then the latter’s hepatic burden tends to decrease since more is metabolized by the induced CYP1A2 despite the increased sequestration capacity. In summary, the extrahepatic disease risk of mixtures may be lower than estimated based on linear summation of single exposures.

TEQ is an important metric that is used to evaluate the toxicity potentials and contributions of DLCs and their mixtures. Traditionally, the TEQ values are calculated for oral exposures using TEF assigned to each DLC congener. Since the TEF values were mostly obtained based on in vivo rodent assays, inter-species uncertainties exist when applied to humans. Rodents and humans are quite different in the toxicokinetics of dioxins, where dioxins are metabolized significantly slower in humans. For mixture exposures, congener–congener interactions can also play a significant role in the tissue burden and overall toxicity. Therefore, it is recommended that if human plasma or tissue concentrations are available, as in biomonitoring data, the body burden TEQ can be calculated directly. However, applying traditional “intake” TEF values to blood or tissue concentrations can lead to miscalculated mixture risk, due to the toxicokinetic differences between species and potential toxicokinetic interactions between congeners [37,68,69,70,71]. Therefore, using systemic TEF values, obtained from in vitro assays, on blood or tissue concentrations is more appropriate [37]. For example, HpCDD and a few other DLCs have significantly different systemic TEF values than the WHO-TEF values. Using the mixture PBPK model, we showed that the contributions of individual congeners to tissue TEQ can be quite different to intake TEQ, depending on the toxicokinetic interactions of the mixture. The relative contributions also vary between blood and different tissues. A congener’s contribution to the TEQ of an extrahepatic tissue, such as fat or muscle, is positively correlated with its partition coefficient for the tissue. In contrast, the binding affinity for CYP1A2 determines the contribution of a congener to the hepatic TEQ. In our mixture model, since all congeners share the same binding affinity for CYP1A2, their relative contribution to hepatic TEQ are nearly identical to blood TEQ since the amount of a congener sequestered in the liver is proportional to its plasma concentration.

In conclusion, we have developed a coding framework to facilitate PBPK modeling for DLC mixtures. The modeling tool is freely available, and as the chemical-specific parameters of congeners are better informed, the refined model can be used for DLC mixture risk assessment in the future.

## Data Availability

Not applicable.

## Supplementary Materials

The following supporting information can be downloaded at: https://www.mdpi.com/article/10.3390/toxics10110700/s1, Table S1: ODEs; Table S2: Physiological and chemical parameters; Table S3: DLC-specific parameters; Table S4: DLC dose sampling parameters.
