# Supplementary material for: A Physiologically Based Pharmacokinetic (PBPK) Modeling Framework for Mixtures of Dioxin-like Compounds
Source: Toxics. 2022 Nov 17;10(11):700. doi: 10.3390/toxics10110700 (PMC9698634; doi:10.3390/toxics10110700)
Supplement: Supplementary file 1 [file toxics-10-00700-s001.zip › Table S1 - ODEs.pdf]

Table S1. ODEs of the Human DLC Mixture PBPK Model

1	Amount in GI track	$\frac{dMST}{dt}$	=	$-(KST + KABS)\odot MST + ORAL\_DOSE\_RATE$
2	Amount absorbed into lymph circulation	$\frac{dLYMLUM}{dt}$	=	$KABS\odot MST\odot a$
3	Amount absorbed into portal circulation	$\frac{dLIMLUM}{dt}$	=	$KABS\odot MST\odot b$
4	Amount of urinary excretion	$\frac{dAURI}{dt}$	=	$CLURI\odot CB$
5	Amount in fat blood	$\frac{dAFB}{dt}$	=	$QF*(CA - \frac{AFB}{VFB}) - PAF\odot(\frac{AFB}{VFB} - \left(\frac{AF}{VF}\right)\odot\frac{1}{PF})$
6	Amount in fat tissue	$\frac{dAF}{dt}$	=	$PAF\odot(\frac{AFB}{VFB} - (\frac{AF}{VF})\odot\frac{1}{PF})$
7	Amount in RB blood	$\frac{dARBB}{dt}$	=	$QRB*(CA-\frac{ARBB}{VRBB}) - PARB\odot(\frac{ARBB}{VRBB} - \frac{ARB}{VRB}\odot\frac{1}{PRB})$
8	Amount in RB tissue	$\frac{dARB}{dt}$	=	$PARB\odot(\frac{ARBB}{VRBB} - \frac{ARB}{VRB}\odot\frac{1}{PRB})$
9	Amount in liver blood	$\frac{dALB}{dt}$	=	$QL*(CA-\frac{ALB}{VLB}) - PAL\odot(\frac{ALB}{VLB} - (\frac{AL}{VL})\odot\frac{1}{PL}) + KABS\odot MST\odot b$
10	Amount of free and nonspecific bound in liver tissue	$\frac{dAL}{dt}$	=	$PAL\odot(\frac{ALB}{VLB} - (\frac{AL}{VL})\odot\frac{1}{PL}) + (-kelim\odot(\frac{AL}{VL})\odot\frac{1}{PL}*\frac{CYP1A2+\Sigma(\frac{A\_Dioxin\_CYP1A2}{VL})-CYP1A2\_1BASAL}{CYP1A2\_1BASAL} - kfAHR\odot(\frac{AL}{VL})\odot\frac{1}{PL}*(AHRtot - \Sigma(\frac{A\_Dioxin\_AHR}{VL})) + kbAHR\odot\frac{A\_Dioxin\_AHR}{VL} - kf1A2\odot(\frac{AL}{VL})\odot\frac{1}{PL}*CYP1A2 + kb1A2\odot\frac{A\_Dioxin\_CYP1A2}{VL})*VL$
11	Amount of Dioxin-AHR complex	$\frac{dA\_Dioxin\_AHR}{dt}$	=	$(kfAHR\odot(\frac{AL}{VL})\odot\frac{1}{PL}*(AHRtot - \Sigma(\frac{A\_Dioxin\_AHR}{VL})) - kbAHR\odot\frac{A\_Dioxin\_AHR}{VL})*VL$

12	Amount of Dioxin-CYP1A2 complex	$\frac{dA\_Dioxin\_CYP1A2}{dt} = (kf1A2 \odot (\frac{AL}{VL}) \odot \frac{1}{PL} * CYP1A2 - kb1A2 \odot \frac{A\_Dioxin\_CYP1A2}{VL}) * VL$
13	Concentration of CYP1A2 mRNA	$\frac{dCYP1A2\_mRNA}{dt} = ktranscription\_1A2 * (1 + \frac{CYP1A2\_1EMAX * (\Sigma \frac{A\_Dioxin\_AHR}{VL})^{0.6}}{CYP1A2\_1EC50^{0.6} + (\Sigma \frac{A\_Dioxin\_AHR}{VL})^{0.6}}) - kdegCYP1A2\_mRNA * CYP1A2\_mRNA$
14	Concentration of free CYP1A2	$\frac{dCYP1A2}{dt} = ktranslation\_1A2 * CYP1A2\_mRNA - kdegCYP1A2 * CYP1A2 - \Sigma(kf1A2 \odot (\frac{AL}{VL}) \odot \frac{1}{PL} * CYP1A2) + \Sigma(kb1A2 \odot \frac{A\_Dioxin\_CYP1A2}{VL})$
