# Supplementary material for: A Physiologically Based Pharmacokinetic (PBPK) Modeling Framework for Mixtures of Dioxin-like Compounds
Source: Toxics. 2022 Nov 17;10(11):700. doi: 10.3390/toxics10110700 (PMC9698634; doi:10.3390/toxics10110700)
Supplement: Supplementary file 1 [file toxics-10-00700-s001.zip › Table S2 - Physiological and biochemical parameters.pdf]

Table S2. Physiological and Biochemical Parameters of the Human DLC Mixture PBPK Model

Parameter	Symbol	Value or Equation	Unit
Body weight	*BW*	**Male:**<br>0.00058*ageInYears^3 - 0.0948*ageInYears^2<br>+ 4.8434*ageInYears + 2.2785<br>**Female:**<br>0.0006*ageInYears^3 - 0.0912*ageInYears^2<br>+ 4.3200*ageInYears + 3.6520	kg
Tissue volume fraction of *BW*			
Liver	*VLC*	3.59E-2 - 4.76E-7**BW**1E3<br>+ 8.50E-12*(*BW**1E3)^2 - 5.45E-17*(*BW**1E3)^3	-
Fat	*VFC*	**Male:**<br>- 5.26E-20*(*BW**1000)^4 + 1.09E-14*(*BW**1000)^3<br>- 6.99E-10*(BW*1000)^2 + 1.59E-5*BW*1000<br>+ 3.95E-2<br>**Female:**<br>- 6.36E-20*(*BW**1000)^4 + 1.12E-14*(*BW**1000)^3<br>- 5.8E-10*(*BW**1000)^2.0 + 1.2E-5**BW**1000<br>+ 5.91E-2	-
Rest of Body (RB)	*VRBC*	(0.91 - *VLBC***VLC* - *VFBC***VFC* - *VLC* - *VFC*) /<br>(1+*VRBBC*)	-
Tissue blood volume fraction			
Liver	*VLBC*	0.266	-
Fat	*VFBC*	0.05	-
RB	*VRBBC*	0.03	-
Cardiac Output	*QCC*	15.36	L/kg/h
Tissue fractional blood flow of *QCC*			
Liver	*QLC*	0.266	-
Fat	*QFC*	0.05	-
RB	*QRBC*	0.03	-
AHR total	AHR_{tot}	0.35	nM
Basal concentration of CYP1A2	*CYP1A2_1BASAL*	1600	nM
Affinity constant of AHR-mediated CYP1A2 induction	*CYP1A2_1EC50*	130	nM
Maximal fold of CYP1A2 induction	*CYP1A2_1EMAX*	9300	-
Rate constant of CYP1A2 mRNA transcription	$k_{transcription\_1A2}$	16	nM/h

Rate constant of CYP1A2 mRNA degradation	$kdeg_{CYP1A2\_mRNA}$	0.1	1/h
Rate constant of CYP1A2 translation	$k_{translation\_1A2}$	1.25	1/h
Rate constant of CYP1A2 degradation	$kdeg_{CYP1A2}$	0.125	1/h

Note: All parameter values or equations are from Emond et al. 2018, except for $k_{translation\_1A2}$, $kdeg_{CYP1A2}$, $k_{transcription\_1A2}$, and $kdeg_{CYP1A2\_mRNA}$, which were adjusted such that the basal and maximally induced steady-state levels of CYP1A2 remain the same as in the Emond model and the steady-state mRNA level was kept to be 10% of the protein level.
