# Supplementary material for: A Physiologically Based Pharmacokinetic (PBPK) Modeling Framework for Mixtures of Dioxin-like Compounds
Source: Toxics. 2022 Nov 17;10(11):700. doi: 10.3390/toxics10110700 (PMC9698634; doi:10.3390/toxics10110700)
Supplement: Supplementary file 1 [file toxics-10-00700-s001.zip › Table S4 - DLC dose sampling parameters.pdf]

Table S4. Monte Carlo Oral Dosing Parameters

	Mean (ng/kg bw/day)	CV
**2,3,4,7,8-PeCDF**	3.90E-04	0.55
**1,2,3,7,8-PeCDF**	6.00E-05	0.42
**2,3,7,8-TCDF**	3.50E-04	0.7
**1,2,3,4,7,8-HxCDF**	4.60E-04	0.4
**2,3,7,8-TCDD**	6.00E-05	0.35
**1,2,3,6,7,8-HxCDF**	2.30E-04	0.4
**1,2,3,4,6,7,8-HpCDD**	2.50E-03	0.8
**1,2,3,4,6,7,8-HpCDF**	8.60E-04	0.43
**1,2,3,7,8-PeCDD**	8.00E-05	0.47
**1,2,3,4,6,7,8,9-OCDD**	1.95E-02	0.64
**1,2,3,4,6,7,8,9-OCDF**	1.16E-03	0.54

Note: These dietary intake values were estimated for a Dutch population in 1994 (Liem et al. 2000) Each congener's daily exposure dose is sampled from lognormal distributions with the means and coefficients of variation (CV).
